# Deep Characterization of the Microbiomes of *Calophya* spp. (Hemiptera: Calophyidae) Gall-Inducing Psyllids Reveals the Absence of Plant Pathogenic Bacteria and Three Dominant Endosymbionts

**DOI:** 10.1371/journal.pone.0132248

**Published:** 2015-07-10

**Authors:** Will A. Overholt, Rodrigo Diaz, Erin Rosskopf, Stefan J. Green, William A. Overholt

**Affiliations:** 1 School of Biology, Georgia Institute of Technology, Atlanta, Georgia, United States of America; 2 Department of Entomology, Louisiana State University, Baton Rouge, Louisiana, United States of America; 3 United States Horticultural Research Laboratory, United States Department of Agriculture, Fort Pierce, Florida, United States of America; 4 Department of Biological Sciences, University of Illinois at Chicago, Chicago, Illinois, United States of America; 5 Biological Control Research and Containment Laboratory, University of Florida, Fort Pierce, Florida, United States of America; University of Vienna, AUSTRIA

## Abstract

Bacteria associated with sap-feeding insect herbivores include not only symbionts that may increase their hosts’ fitness but also harmful plant pathogens. *Calophya* spp. gall-inducing psyllids (Hemiptera: Calophyidae) are being investigated for their potential as biological control agents of the noxious weed, Brazilian peppertree (*Schinus terebinthifolia*), in Florida. Although there are no examples of plant pathogen transmission by members of the family Calophyidae, several insects in the superfamily Psylloidea are known to transmit pathogenic bacteria in the genera *Candidatus* Liberibacter and *Candidatus* Phytoplasma. To determine whether *Calophya* spp. harbor potentially harmful plant pathogenic bacteria, we sequenced small subunit (SSU) ribosomal RNA (rRNA) gene amplicons generated from individuals from four *Calophya* spp. populations. All microbial SSU gene sequences fell into the bacterial domain, with 98-99% belonging to the Proteobacteria. The *Calophya* microbiomes contained a relatively simple community, with 49-79 operational taxonomic units (OTUs; 97%) detected, and only 5-8 OTUs with greater than 1% abundance. *Candidatus* Carsonella showed the highest relative abundance, with OTUs from this candidate genus representing between 51 – 65% of all recovered sequences. The next most abundant clade observed was an unclassified Enterobacteriacae group closely related to bacteria from the genera *Buchnera* and *Blochmannia* that ranged from 20-31% in relative abundance. *Wolbachia* populations were the third most abundant group and represented 7-27% of the diversity in microbial OTUs. No SSU rRNA gene sequences from putative pathogenic bacteria from the genera *Ca*. Liberibacter or *Ca*. Phytoplasma were detected in the microbiomes of the four *Calophya* populations. The probability that infected psyllids were present in our colonies, but were not sampled, was extremley low (1.39 x 10^-10^). As far as we are aware, our study is the first to characterize the microbiome of a candidate biological control agent, and coupled with previous work demonstrating a high degree of host specificity and absence of plant viruses, suggests that releasing *Calophya* spp. in United States poses minimal risk to non-target plants.

## Introduction

After habitat destruction, competition with invasive species is considered the greatest threat to imperiled native flora and fauna in the USA [1]. Within the continental USA, Florida is most vulnerable to invasion due to its island-like biogeography, high level of disturbance, and the incessant introduction of exotic species [2]. Of the 4,304 plants species that reproduce outside of cultivation in Florida, 1,440 (33%) are exotic [3] and 157 of these are considered invasive by the Florida Exotic Pest Plant Council [4]. Among the invasive plants, Brazilian peppertree (*Schinus terebinthifolia*) stands out as one of the most problematic, having invaded an estimated 283,000 ha of the state [5] where it severely reduces native community diversity [6]. Herbicides and mechanical control are the standard methods for management of Brazilian peppertree [7], but these tactics are costly [5], can harm non-target vegetation [8], and repeated treatments are required to prevent regrowth [5].

Classical biological control of Brazilian peppertree has been pursued for several years, but no natural enemies have yet been approved for release in the continental United States [9]. Recent foreign exploration in the coastal regions of Brazil has led to the discovery of leaf galling psyllids in the genus *Calophya* (Hemiptera: Calophyidae) attacking Brazilian peppertree [10,11]. Based on field collections, the *Calophya* spp. found on Brazilian peppertree have narrow host associations and in some cases appear to be monophagous [12,13]. *Calophya* spp. collected from Brazilian peppertree at four locations in Brazil were imported into Florida quarantine for further examination, including two populations of *Calophya latiforceps* Burckhardt from Bahia and Espiritu Santo, one undescribed *Calophya* sp. from Espirito Santo, and *Calophya terebinthifolii* Burckhardt and Basset from Santa Catarina. *Calophya latiforceps* completes one generation in 39 d with 40% survival. Gall initiation and growth resulted in yellowing, deformation and abscission of Brazilian peppertree leaves [14]. Host specificity tests demonstrated that *C*. *latiforceps* nymphs can induce galls and complete development only on Brazilian peppertree and those adults exposed to non-target plants only had greatly reduced survival [15].

In spite of the promising biological control potential of *Calophya* spp., many species in the superfamily Psylloidea are known vectors of plant pathogenic bacteria including Gram-negative *Candidatus* Liberibacter [16] and Gram-positive *Candidatus* Phytoplasma [17]. The transmission of members of *Ca*. Liberibacter by the Asian citrus psyllid (*Diaphorina citri* Kumayama, Psyllidae), carrot psyllid (*Trioza apicalis* Förster, Triozidae), and potato psyllid (*Bactericera cockerelli* Sulc., Triozidae) has resulted in significant economic losses in citrus [18], carrots, and potatoes [19,20], respectively. Species of *Ca*. Phytoplasma cause more than 700 diseases and have been reported in the Psyllidae genera *Cacopsylla* and *Bactericera* [17,21]. Insect vectored pathogenic bacteria may also have positive effects. *Ca*. Liberibacter europaeus, vectored by the psyllid, *Arytainilla spartiophila* (Föerster) (Psyllidae), may help suppress populations of the noxious weed, Scotch broom (Fabales: Fabaceae: *Cytisus scoparius*) in Europe and New Zealand [22].

The microbiomes of agricultural pest pysllids have been well characterized, initially with Sanger sequencing of cloned amplicons of the small subunit (SSU) rRNA gene, and more recently using next generation sequencing approaches [23–27]. The microbiomes of phloem feeding insects are typically characterized by a very low diversity of <10 bacterial operational taxonomic units, and for psyllids, are composed of the primary endosymbion**t** *Ca*. Carsonella and several secondary endosymbionts [25]. The vast majority of these studies focused on the microbiome of the potato psyllid (*Bactericera cockerelli*) and the Asian citrus psyllid (*Diaphorina citri*) [23,24,27]. Only one study has investigated bacteria associated with *Calophya* (*Calophya schini*), and this study only generated two SSU rRNA gene sequences from *Calophya schini*, one for *Ca*. Carsonella and a secondary endosymbiont associated with the *Enterobacteriaceae* [28,29].

Previously, we were unable to detect bacteria in *Calophya latiforceps* from the species *Ca*. Liberibacter solanacearum, *Ca*. L. asiaticus, *Ca*. L. americanus or *Ca*. L. africanus in amplification reactions employing species-specific primer sets [30]. We also found no evidence of plant viruses in *C*. *latiforceps* using both molecular and inoculation techniques [30]. Although targeted polymerase chain reaction (PCR) amplification reactions are highly sensitive to low levels of target deoxyribonucleic acid (DNA), the specificity of the assays can miss closely related taxa of interest. Furthermore, extensive evidence of associations between psyllids and plant pathogenic bacteria prompted us to employ a broader approach to detect potentially pathogenic bacteria. Therefore, the goal of this study was to characterize the entire microbial community present in several *Calophya* species. To achieve this, we performed ultra-deep high-throughput sequencing of SSU rRNA gene amplicons generated from genomic DNA (gDNA) extracted from *Calophya*. These results are useful in determining not only the potential safety of these candidate biological control agents but also provide new insight into the diversity of bacterial symbionts associated with psyllids. As far as we are aware, this is the first example of complete characterization of the microbiome of a candidate weed biological control agent.

## Materials and Methods

### Insect collection

Adults of *Calophya* spp. were obtained from colonies maintained at the biological control quarantine facility of the University of Florida in Fort Pierce, Florida. These colonies were initiated using adults collected from galled leaves at locations along coastal Brazil from 2012 to 2014 under permit no. 12BR008156/DF issused to W. A. Overholt from the Brazilian Institute of Environment and Renewable Natural Resources. The colonies included two populations of *Calophya latiforceps*, one population of *Calophya terebinthifolii* and one population of an undescribed *Calophya* species.

The designations used for the four *Calophya* populations used in this study, along with their origins are included in Table 1. Upon arrival to the quarantine facility, newly emerged adults from each location were reared on Brazilian peppertree saplings that were grown out-of-doors. In May 2014, three groups of 20 adults ranging in age from newly emerged to approximately 15 days old (maximum longevity [14]) were haphazardly collected from each population and placed at -20°C until processing.

**Table 1:** Colony designations, species, origins and Florida State Collection of Arthropods voucher numbers of *Calophya* spp. examined for microbiome diversity.

Designation	Species	Origin in Brazil	Voucher no. Florida State Collection of Arthropods
Salvador	*Calophya latiforceps*	12.908°S, 38.336°W Salvador, Bahia State	E2013-3192-1
*C*. *tere*	*Calophya terebinthifolii*	26.921°S, 48.640°W Balneario Camboriu, Santa Catarina State	E2014-5749-1
Carapina	*Calophya latiforceps*	20.213°S, 40.229°W, Carapina, Espiritu Santo State	E2013-6744-1
Ubu	undescribed *Calophya* sp.	20.786°S, 40.579°WUbu, Espiritu Santo State	E2013-6743-1

### DNA extraction and amplification

Psyllid DNA was extracted from three sub-samples of each population, using the DNeasy Blood & Tissue Kit (Qiagen, Venlo Netherlands) as reported for the extraction of DNA from the Asian citrus psyllid, *Diaphorina citri* [31]. DNA from twenty psyllids (a group) was extracted using the manufacturer’s instructions. Extracted DNA was stored at -20°C until used for PCR amplification.

### Characterization of the psyllid microbiome

Genomic DNA was used as input for PCR amplification of the V4 variable region of microbial SSU rRNA genes using indexed primers as described previously [32]. Primers were assessed *in silico* using the Probe Match program associated with RDP and the program Pimer Prospector against our database [33,34]. These primers hit 99% of all high quality *Ca*. Phytoplasma sequences in the RDP database when allowing 1 mismatch (92% perfectly), and 100% of *Ca*. Phytoplasma sequences in our database.

After equalizing PCR yields, PCR products were pooled and cleaned. Amplification and sequencing were performed at the RTSF Genomics Core at Michigan State University. Pooled products were loaded on a standard MiSeq flow cell (v2) and sequenced in a paired end 2x250bp format. The resulting sequences were processed in QIIME v.1.8 [35]. Briefly, overlapping reads were merged using fastq-join [36], and quality filtering was performed using USEARCH v7.0 where reads with an expected error >0.5 were excluded from further analysis [37]. Only completely assembled reads of approximately 250 bases (after primer sequence removal) were utilized for further analysis. Operational taxonomic units (OTUs) were defined at 97% sequence similarity using USEARCH v.7.0 [37] and chimeras were removed *de novo* following the UPARSE pipeline and by comparing against representative sequences from the Greengenes database v.13_8 [38] clustered at 91% sequence identity to minimize computational time. OTUs were also defined at 99% sequence similarity using USEARCH v.7.0 as described above to determine if dominant OTUs from the populations originating from Salvador, Carapina, and Ubu could be distinguished.

A representative set of OTU sequences were classified using the Ribosomal Database Project classifying algorithm [33] that had been re-trained with representative sequences from the Greengenes database v.13_8. Sequences derived from plastids (chloroplast or mitochondria) were discarded from further analysis. Sequences were aligned using the PyNAST aligner [39] and the Greengenes v.13_8 database. Any sequence that failed to align was discarded from further analysis. Raw sequence data (FASTQ files) were deposited in the NCBI Sequence Read Archive under project SRP049390.

### Statistical analysis

The QIIME generated OTU table was imported into R v.3.1.1 [40] and sampling effort was adjusted to a common scale by calculating size scaling factors using the R package DESeq2 [41]. Other normalization and transformation approaches were tested including using cumulative-sum scaling with the R package ‘metagenomeSeq’ [42], and subsampling to 100,000 sequences per library (S1 Fig). Shannon diversity for each sample was calculated from the DESeq2 adjusted OTU table using the package ‘Vegan’ [43]. An ANOVA was performed to test for differences in the means of the Shannon values, followed by Student-Newman-Keuls post-hoc means separation procedure (P < 0.05).

Rarefaction curves were generated using the ‘rarefy’ function in ‘vegan’ by repeatedly subsampling the OTU table at steps of 1000. A mean number of OTUs and the standard deviation for each sample per step was calculated using the R package ‘plyr’ and plotted using the R package ‘ggplot2’ [44,45]. Total richness was estimated using the R package ‘preseqR’ [46] by fitting a zero-truncated negative binomial model and extending to 100 million sequences per sample.

Two dominant classes for all microbiomes were identified, and the top OTUs belonging to these dominant classes were determined. OTU sequences were placed onto a pre-compiled SSU rRNA gene tree maintained by The Greengenes Database Consortium containing 408,000 full length Bacterial and Archaeal gene sequences using ARB [47]. Similar reference sequences within the database were selected and the phylogenetic tree was exported and visualized using iTOL [48].

To compare microbial community structures, all OTU counts were transformed using a variance stabilizing approach implemented in DESeq2 and recommended by McMurdie and Holmes [49]. A Bray-Curtis similarity matrix was calculated using the package ‘vegan’ in R [43] and similarities were visualized using a nonmetric multidimensional scaling plot (NMDS) generated using the R package ‘ggplot2’ [44]. Confidence regions (95%) for each group were calculated using ‘vegan’s’ oridellipse function and overlaid on the NMDS plot to estimate the area within the ordination space that contains the true value. Insect microbiome groupings (replicates) were statistically verified using an adonis test (R package ‘vegan’) and a SIMPROF test within the software package Primer-E v.6 [43,50,51].

### Screening for potential plant pathogens

Initially, the RDP assigned taxonomy was screened for OTUs assigned to *Ca*. Liberibacter or *Ca*. Phytoplasma genera. After verifying that no OTU was annotated to either of these groups, a reference set of full length SSU rRNA gene sequences from each group was generated from sequences submitted to GenBank. The initial dataset included 34 *Ca*. Liberibacter and 589 *Ca*. Phytoplasma sequences of greater than 1400 bases. Reference sequences were screened phylogenetically to remove mis-annotated sequences (e.g. EU093079.1 –*Ca*. Phytoplasma RYL-GD, S2 Fig). An additional 3 of the sequences failed to align using the PyNAST aligner and were also excluded, yielding a total of 573 *Ca*. Phytoplasma sequences. Using nucleotide BLAST v.2.2.28+, all quality controlled amplicon sequences generated in this study were compared against this reference database (this database is provided in the supporting information). A positive detection for either group was considered >94% similarity across 80% of the sequence read.

## Results

### Characterization of the *Calophya* microbiome

**Fig 1:**
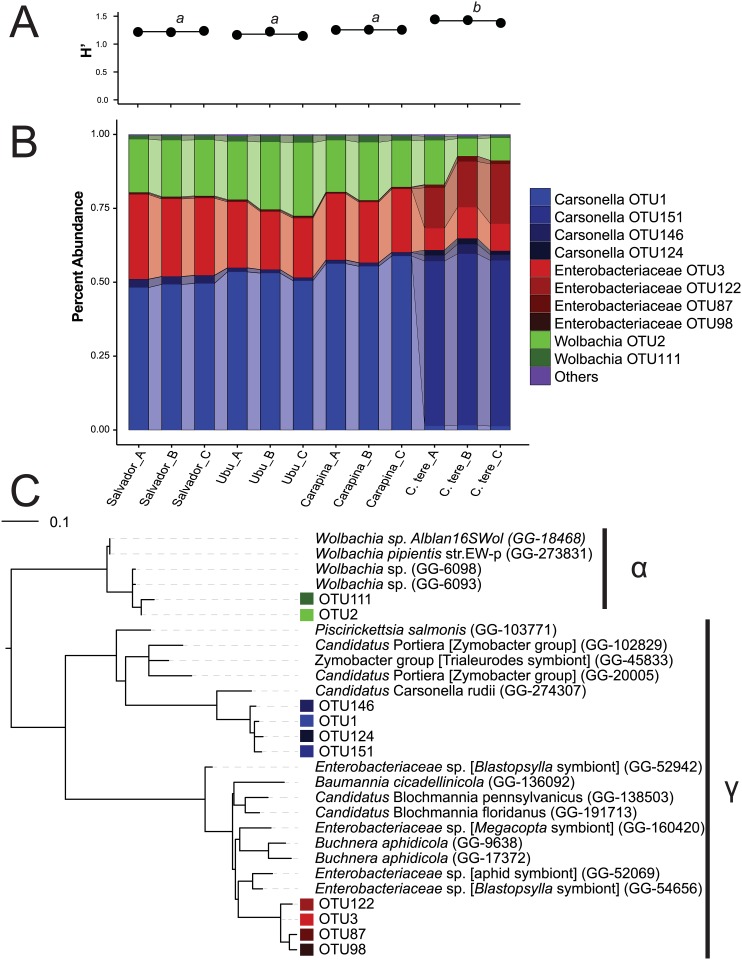
The diversity, composition, and phylogeny of dominant members of the *Calophya* spp. microbiome. (A) Shannon diversity indices for each microbiome sampled. The average value for each *Calophya* sp. (run in triplicate) is indicated with a line. Superscripts indicate groups that are statistically different by pairwise t-tests. (B) Microbial community composition for each microbiome analyzed. OTU abundances were estimated by converting libraries to a common scale using DESeq2, and normalized using total counts. OTUs from each dominant clade are colored with the same hue, and in all cases these dominant clades represent >98% of the total community. OTU bars are connected with semi-transparent edges to make it easier to distinguish between different OTUs within the same group. (C) Dominant OTUs were inserted into a pre-compiled phylogenetic tree using ARB. OTU are labeled using the same color scheme as in (B).

**Fig 2:**
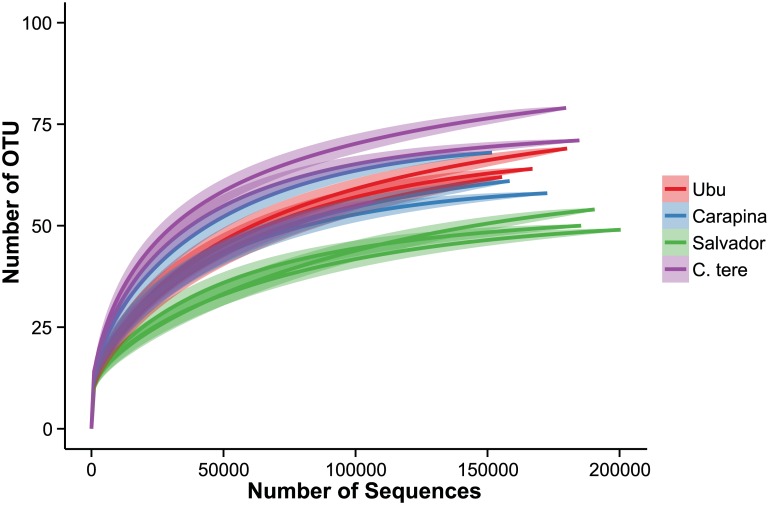
The microbial community richness sampled from *Calophya* spp. Rarefaction curves generated in R using the package ‘vegan’. Standard deviations for each step are indicated with the semi-transparent shading.

The microbiomes of the four populations of *Calophya* spp., in triplicate, were determined by deep sequencing of microbial SSU rRNA gene amplicons generated from total insect DNA extracts (10 individuals per replicate sample). In total, over 2 million sequences were generated from 12 samples. All sequences passing quality control filters were derived from organisms within the domain Bacteria, with greater than 98% of the sequences derived from organisms within the phylum Proteobacteria. The microbiomes of all tested organisms were highly similar, had low overall species richness with 49–79 detected OTUs, and housed few dominant taxa (Fig 1). Only 5 OTUs had >1% abundance in populations from Salvador, Ubu, and Carapina, while 8 OTUs >1% were present in *C*. *terebinthifolii* (Table 2). Approximately 80% of the total richness was sampled as estimated using a fitted zero-truncated negative binomial model at 10 million sequences per sample. Rarefaction curves indicated that the majority of these OTUs were extremely low in abundance (Fig 2). Shannon diversity indices (base e) ranged from 1.16 to 1.44, and the microbial community sampled from *C*. *terebinthifolii* was significantly more diverse than the other three populations (Fig 1). The Salvador population was more diverse than Carapina, but Salvador was not different from Ubu and Ubu was not more diverse than Carapina ((P < 0.05 for all comparisons).

**Table 2:** Sequencing statistics for *Calophya* spp. microbiomes.

SampleID^a^	Sequences Retrieved^b^	Number of OTUs^c^	Estimated Richness^d^	Shannon (H')	Num OTU > 1% Abundance
Salvador A	185424	50 (45)	65 [77%]	1.25 (1.25)	5 (5)
Salvador B	190544	54 (46)	66.46 [81%]	1.26 (1.26)	5 (5)
Salvador C	200392	49 (43)	61.52 [80%]	1.26 (1.25)	5 (5)
Ubu A	180116	69 (59)	85.82 [80%]	1.22 (1.22)	5 (5)
Ubu B	167006	64 (53)	77.08 [83%]	1.21 (1.22)	5 (5)
Ubu C	155472	62 (56)	81.51 [76%]	1.24 (1.24)	5 (5)
Carapina A	172604	58 (55)	79.69 [73%]	1.16 (1.17)	5 (5)
Carapina B	158362	61 (60)	87.45 [70%]	1.22 (1.22)	5 (5)
Carapina C	151683	68 (63)	92.16 [74%]	1.15 (1.15)	5 (5)
*C*. *tere*_A	184772	71 (63)	91.68 [77%]	1.44 (1.45)	8 (8)
*C*. *tere*_B	179729	79 (69)	101.33 [78%]	1.43 (1.44)	8 (8)
*C*. *tere*_C	107640	56 (56)	81.29 [69%]	1.38 (1.38)	8 (8)

^a^Salvador and Carapina belong to *C*. *latiforceps*, Ubu refers to an undescribed *Calophya* species, and *C*. *tere* refers to *C*. *terebinthifolii* (see Table 1). Values in parentheses were calculated using rarefied libraries to 100,000 sequences per sample, values before parentheses were calculated using DESeq2 normalized OTU counts.

^b^Number of sequences after quality control.

^c^OTU defined at 97% sequence similarity.

^d^Predicted using a fitted zero truncated negative binomial model to 100 million sequences per sample for rarefied data only. Values in brackets are %coverage using DESeq2 normalized OTU counts.

**Fig 3:**
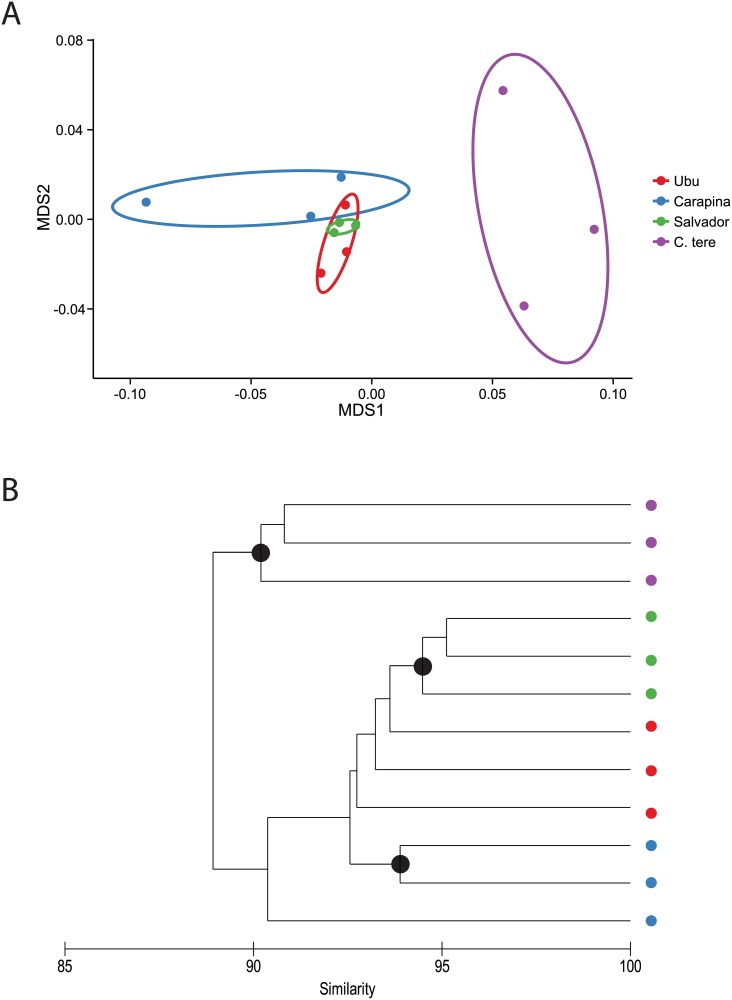
Comparing microbial communities associated with *Calophya* spp. (A) *Calophya* microbiomes were compared by computing Bray-Curtis similarities using variance stabilized OTU counts, and visualized using a non-metric multidimensional scaling plot (stress = 0.09). 95% confidence ellipses are shown, and *C*. *terebinthifolii* confidence ellipses clearly do not overlap with the other *Calophya* spp. (B) A SIMPROF test was performed and visualized using Primer-6. Black circles indicate groups that cannot be significantly differentiated. Again, *C*. *terebinthifolii* forms a cohesive group distinguishable from the other three *Calophya* spp. which cannot be separated into distinct groups.

The microbial community structures for all *Calophya* spp. were compared using the Bray-Curtis diversity index, which uses the microbial populations present and their relative abundances. A relatively extreme transformation, variance stabilization approach using the R package ‘DESeq2’, was applied, similar to what was observed with a log (X+1) transformation (S1 Fig). Transformation choice played a relatively important role in distinguishing between the populations from Salvador, Ubu, and Carapina. In all cases, a nonparametric ANOVA (adonis) test revealed sample groups were significant (R^2^ >0.90, P< 0.01), but these tests do not have enough power to resolve post-hoc pairwise testing. However, *C*. *terebinthifolii* was significantly different from the other three populations based on non-overlapping 95% confidence ellipsoids and a SIMPROF test with 9999 permutations, independent of the normalization and transformations that were applied (Fig 3A and 3B, S1 Fig). Conversely, *Calophya latiforceps*-Salvador could only be distinguished from Ubu and Carapina as an internal cluster (with non-overlapping 95% confidence ellipsoids) after applying a strong transformation. Using the R package ‘metagenomeSeq’ to normalize libraries to a common scale (using cumulative-sum scaling to the 75^th^ percentile) [42], we can distinguish between *C*. *latiforceps*-Salvador and the other two populations.

The microbiome of *C*. *terebinthifolii* was highly distinct from that of all other *Calophya* species tested. Nonetheless, the *C*. *terebinthifolii* microbiome contained dominant taxa that were closely related to the dominant taxa in other species. Bacteria from the candidate genus *Ca*. Carsonella were the most abundant in all *Calophya* tested, with four OTUs representing between 51–65% of rRNA gene amplicon sequences from each sample. Two dominant OTUs within the *Ca*. Carsonella clade were detected. *Ca*. Carsonella OTU_1 was present in all samples but was highly enriched in *C*. *latiforceps* (Salvador and Carapina) and *Calophya* sp. (Ubu) relative to *C*. *terebinthifolii*. The *C*. *terebinthifolii* microbiome was dominated by a distinct *Ca*. Carsonella population, OTU_151, which was not detected in the other three insect populations (Fig 1B). The next most abundant clade, consisting of four OTUs, was affiliated with an unclassified Enterobacteriacae group (Gammaproteobacteria) similar to *Buchnera* and *Blochmannia* (90–94% sequence identity) that ranged from 20–31% abundance (Fig 1B and 1C). Enterobacteriacae OTU_87 was more abundant in *C*. *terebinthifolii*, while OTU_3 was enriched in the other three insect microbiomes. *Wolbachia* (Alphaproteobacteria) taxa were detected in all microbiomes ranging in abundance from 7–27%, and were primarily represented by a single OTU (OTU2). Overall the *C*. *terebinthifolii* microbiome had a lower abundance of *Wolbachia* sequences than the microbiome of other *Calophya* species, although there was substantial variation between the *C*. *terebinthifolii* replicates (Figs 1B and 3A).

### Absence of pathogenic bacteria

A database of reference sequences of disease causative agents in the genera *Ca*. Liberibacter and *Ca*. Phytoplasma was generated that contained 603 high quality, phylogenetically verified, >1400bp sequences (Supporting Information). All 2 million quality controlled SSU rRNA gene amplicon reads were aligned to the reference database using blastn [52], retaining any hits >94% sequence similarity for at least 200 bases (~80% of the sequence). No sequences met these criteria, out of a total of 2,077,290 sequences. The closest sequences were 93% similar to *Ca*. Liberibacter crescens (Genbank accession number NR_102476); however, they were 94% similar to *Rhizobium leguminosarum* (AY509900), and 99% similar to an unclassified *Rhizobium* (JQ977368). These *Rhizobium* and *Ca*. Liberibacter strains were approximately 92% similar to each other across nearly full length SSU rRNA gene sequences. This analysis demonstrated that there were no detectable populations closely related to *Ca*. Liberibacter or *Ca*. Phytoplasma in the *Calophya* microbiomes examined in this study.

## Discussion

### Phylogeny of bacteria and *Calophya*

The objectives of this study were to determine if *Calophya* species harbor plant pathogenic bacteria, and more broadly to assess the whole-body microbiome of these promising biocontrol agents. Despite an extremely deep SSU rRNA gene amplicon sequencing effort, no pathogens were detected, and we conclude that releasing *Calophya* species as biocontrol agents of Brazilian peppertree poses no risk of introducing plant pathogenic bacteria.

Overall, the *Calophya* microbiome of multiple species was found to be extraordinarily limited, consistent with prior studies of psyllid microbiomes [25]. Specifically, all four populations of *Calophya* spp. contained the primary obligate endosymbiont *Ca*. Carsonella [53]. *Ca*. Carsonella is considered a primary endosymbiont of Psylloidea, with a role in synthesizing essential nutrients required by their sap-feeding hosts [28,54]. Recent work has shown that the highly degraded genome of *Ca*. Carsonella is complemented in amino acid synthesis by genes from secondary endosymbionts [55] and host genes, some of which may have been gained through horizontal transfer from *Ca*. Carsonella [56]

As expected, our results are consistent with the assumption of co-diversification of *Ca*. Carsonella and the host *Calophya* species. A phylogenetic reconstruction of *Calophya* using the mitochondria cytochrome c oxidase I (MT-COI) gene revealed that *C*. *terebinthifolii* was 13–14% divergent from the other three *Calophya* populations [15]. This corresponded to a 3% sequence divergence in the V4 region of the SSU rRNA gene for the *Ca*. Carsonella populations. Higher similarities between the SSU rRNA genes relative to the MT-COI genes were expected due to the high degree of sequence conservation in the SSU rRNA gene [28,57]. MT-COI analysis further revealed a 6% sequence divergence between *C*. *latiforceps*, and *C*. sp. Ubu, which along with morphological differences, was sufficient to warrant further investigation into describing a new species [15]. However, the dominant *Ca*. Carsonella populations retrieved from *C*. sp. Ubu were >99% identical to the ones found in *C*. *latiforceps*. We believe it is unlikely that *Ca*. Carsonella has been shared across matrilines [58], and this pattern is due to the lower resolving power in short SSU rRNA gene fragments [28]. Finally, the *Ca*. Carsonella population from *C*. *latiforceps* was indistinguishable from the one retrieved from Carapina, which was unsurprising since the host populations were also indistinguishable using MT-COI [15].

The majority of psyllids studied to date have contained one or many co-primary symbionts [59,60]. *Ca*. Carsonella species have a highly reduced genome that does not contain sufficient amino acid biosynthesis pathways to meet the needs of the host, and previous studies have shown these functions can be complemented by an obligate secondary endosymbiont [61], or possibly by incorporation of symbiont genes into the host genome [55,61,62]. In our study, all populations of *Calophya* contained sequences derived from bacteria of the broadly distributed family Enterobacteriaceae. Closely related and described strains include the primary symbionts *Blochmannia* from ants, and *Buchnera* from aphids [28,63,64]. Both *Blochmannia* and *Buchnera* have demonstrated roles in nutrient provision [65–68]. Sequences derived from Enterobacteriaceae in this study were highly similar (94–98% identity) to a sequence isolated from *Calophya schini* Tuthill [28], an insect which feeds on a congener of Brazilian peppertree, *Schinus molle* L.

While a definitive phylogenetic tree for the genus *Calophya* has not been conclusively generated, based on morphology, *C*. *schini* is an outgroup to the *Calophya* spp. used in this study [10,11]. This taxonomy is consistent with the divergent microbiome of *C*. *terebinthifolii*. This divergent microbiome included a distinct dominant *Ca*. Carsonella OTU and a distinct Enterobacteriaceae OTU (>3%) that was absent from the other *Calophya* populations. Furthermore, the three *Calophya* populations with indistinguishable *Ca*. Carsonella populations also had indistinguishable dominant Enterobacteriaceae populations (>99% similar). Interestingly, *C*. *terebinthifolii* maintained the distinct Enterobacteriaceae group and the shared Enterobacteriaceae group. We hypothesize that this was due to horizontal re-acquisition of the Enterobacteriaceae strain present in the other three populations, as horizontal transfers of secondary symbionts have been shown to occur [28]. However, we cannot verify that both Enterobacteriaceae populations were present in all *C*. *terebinthifolii* individuals, as each replicate was composed of a mixture of 10 insects. Hypothetically, this same pattern would emerge if approximately 30% of *C*. *terebinthifolii* insects contained only the more typical Enterobacteriaceae population, and ca. 70% contained only the divergent strain.

The final dominant microbial population detected was affiliated with the Alphaproteobacterial genus, *Wolbachia*. *Wolbachia* are a well-studied and diverse group of intracellular bacteria that infect arthropods and nematodes. *Wolbachia* infect more than 65% of insect species, where they are generally considered reproductive parasites because they manipulate host reproduction to favor their spread. This is accomplished through several mechanisms including cytoplasmic incompatiblity, induction of parthenogenesis, feminization and male killing (reviewed in [69]). In addition, *Wolbachia* has been shown to play a mutualistic role in *Drosophila melanogaster* by increasing resistance to RNA viruses [70]. The role of *Wolbachia* in *Calophya* spp. is unknown, but parthenogenesis, feminization and male killing can probably be ruled out, as there has been no evidence for these effects in the established colonies at the University of Florida. Bacteria from the genus *Wolbachia* typically do not share phylogenetic congruencies with their host, and indeed in this study we observed one dominant *Wolbachia* population in all *Calophya* species. This is similar to other studies of psyllid endosymbionts [25,28,71]

Using the relationships between the entire *Calophya* microbiomes, we also observe a strong congruency to the phylogeny of *Calophya*. The 13–14% divergence in MT-COI genes between *C*. *terebinthifolii* corresponds to an average Bray-Curtis dissimilarity value of 11% using a variance stabilizing transformation (78% using untransformed data). This was expected since *Ca*. Carsonella represented ~50% of the total microbiome for each *Calophya* sp. Hence, utilizing both the similarities between microbial community structures and individual endosymbiont populations, we were still incapable of distinguishing between *Calophya latiforceps* and *Calophya* sp. Ubu.

### Reduced bacterial diversity in phloem-feeding insects

Our results provide support for the hypothesis that sap-feeding Hemipterans have low bacterial diversity compared to other insects [25]. Using SSU rRNA gene amplicon sequencing, we detected between 5 and 8 OTUs with an abundance of >1% and an average Shannon index (H’, log e) of 1.27 (range = 1.16–1.44) per *Calophya* sample. In contrast, non-sap-feeding Hemipterans have larger numbers of bacterial OTUs per sample including Hymenoptera (11 OTUs), Lepidoptera (32 OTUs), and Isoptera (90 OTUs) [72]. Our OTU numbers and Shannon indices are consistent with those reported for the potato psyllid, *B*. *cockerelli* (Triozidae), and the Asian citrus psyllid, *D*. *citri* (Psyllidae), which ranged between 3 and 7 OTUs [25,27,71] and Shanon indices (log e) of 0.58 and 1.18, respectively [25]. Similarly, whiteflies (*Bemisia tabaci* Gennadius, [25]) and aphids (*Acyrthosiphon pisum* Harris, [25]; *Aphis glycines* Matsumura, [73]) have low bacterial diversity. Insects feeding on more complex diets appear to require a more diverse microbiota for digestive purposes. For example, the number of OTUs per sample for xylophagous insects of decaying wood and for detritivores was 103 and 53, respectively [72]. The reason for the low diversity of bacteria found in phloem-feeding Hemipterans is unknown, but could be due to the low variation in physical and chemical composition of sap [74], which might not require an association with a large number of bacteria for nutritional purposes. Furthermore, plant galls are nutrient sinks [75] which provide an enriched food source for gall-inducing Hemipterans such as *Calophya* spp. This high quality diet may require less microbial processing than typical phloem sap. Future studies could test this hypothesis by comparing the microbiome diversity in non-galling and gall-inducing *Calophya*.

### Absence of plant pathogens in *Calophya* spp.

The psyllid (Psylloidea) families Psyllidae and Triozidae include several members that transmit plant pathogenic bacteria [76], including species in the genera *Ca*. Phytoplasma and *Ca*. Liberibacter [17]. Examples include Asian citrus psyllid (*Diaphorina citri* Kumayama (Psyllidae) transmission of species of *Ca*. Liberibacter that cause Huanglongbing in *Citrus* spp., and carrot yellows decline transmitted by *Trioza apicalis* Förster and *Bactericera trigonica* Hodkinson (Triozidae) [19,20]. *Ca*. Phytoplasma diseases include European stone fruit yellows (*P*. *prunorum*), transmitted by *Cacopsylla pruni* Scopoli [77] and pear decline (*P*. *pyri*) transmitted by *Cacopsylla pyricola* (Förster) [78]. Although we have not been able to find any records of bacterial plant pathogen transmission by species in the family Calophyidae, we examined *Calophya* to determine whether any plant pathogenic bacteria could be detected. In this study, extraordinarily deep SSU rRNA gene amplicon sequencing (>100,000 sequences per sample) did not reveal any known pathogens. This result confirms prior assays in which specific primers to detect the presence of four species of the genus *Ca*. Liberibacter (*i*.*e*., *Ca*. L. solanacearum, *Ca*. L. asiaticus, *Ca*. L. americanus and *Ca*. L africanus) were negative [15]. The probability that psyllids infected with plant pathogenic bacteria were present in our colonies, but not sampled, can be calculated if we assume a background level of infection. The reported levels of infection by *Ca*. Liberibacter asiaticus, the causitive agent of citrus greening disease, in field collected *D*. *citri* in Florida ranged from 8.75% (Munjanuth et et. 2008) to 81.0% (Ammar et al. 2011). If we conservatively assume the lower level of infection, by assaying 240 psyllids out of an estimated colony size of 4000, the probability that plant pathogenic bacteria were present but not detected was extremely low (1.39 x 10^−10^, hypergeometric test).

Virus transmission by *C*. *latiforceps* is also unlikely as only one member of the superfamily Psylloidea is known to vector a virus [79]. Regardless, we previously tested *C*. *latiforceps* for Tomato torrado virus and Tomato chocolate spot virus using specific primers, and also inoculated highly susceptible indicator plants with homogenized insects, and found no evidence of viruses [30]. Additional evidence of lack of pathogen transmission would be available if we could rear the insects on infected plants to examine acquisition, but host range testing on 89 plant species demonstrated that *C*. *calophya* will only feed on Brazilian peppertree [30]. Thus, we have not been able to determine whether transmission is theoretically possible. Nonetheless, due to the extremely restricted diet and the largely obligate and simple microbiome associated with *Calophya* species, we believe that these organisms are safe to release in the United States.

## Supporting Information

S1 Fig Comparing the effect of OTU table transformations and normalizations on *Calophya* spp. microbiome beta diversity. (A) OTU counts have been normalized using a variance stabilized approach as described in the main methods section. (B) A Log_10_(X+1) transformation on the DESeq2 common scaled OTU table, Love et al. (2014) stated that the variance stabilized normalization was similar to a log normalization [41]. (C) OTU counts have been normalized to a common scale using DESeq2. (D) OTU counts have been normalized using the cumulative-sum scaling method applied with the R package ‘metagenomeSeq’ [42]. (E) OTU counts have been normalized to a common-scale using DESeq2 and square root transformed. (F) OTU counts have been normalized using cumulative-sum scaling and square root transformed. (G) OTU counts have been randomly subsampled without replacement to 100,000 sequences per sample. (H) Raw, non-tranformed and non-normalized, OTU counts. (I) Subsampled OTU counts have been square root transformed. (J) Subsampled OTU counts have been log_10_(X+1) transformed.
(PDF)

S2 Fig Validating the taxonomic annotation of GenBank sequences associated with Phytoplasma used in the reference database. As described in the methods section, the GenBank nucleotide database was screened for any sequence affiliated with the SSU rRNA gene from Phytoplasma spp. All sequences were aligned using PyNAST and a maximum likelihood tree was generated using MEGA v6. Misannotated sequences were excluded from the database.
(PDF)

S1 Dataset Reference database of full length or nearly full length *Liberibacter* and *Phytoplasma* sequences. (FASTA)
