# Supplementary figures and images for: Deep Characterization of the Microbiomes of Calophya spp. (Hemiptera: Calophyidae) Gall-Inducing Psyllids Reveals the Absence of Plant Pathogenic Bacteria and Three Dominant Endosymbionts
Source: PLoS One. 2015 Jul 10;10(7):e0132248. doi: 10.1371/journal.pone.0132248 (PMC4498736; doi:10.1371/journal.pone.0132248)

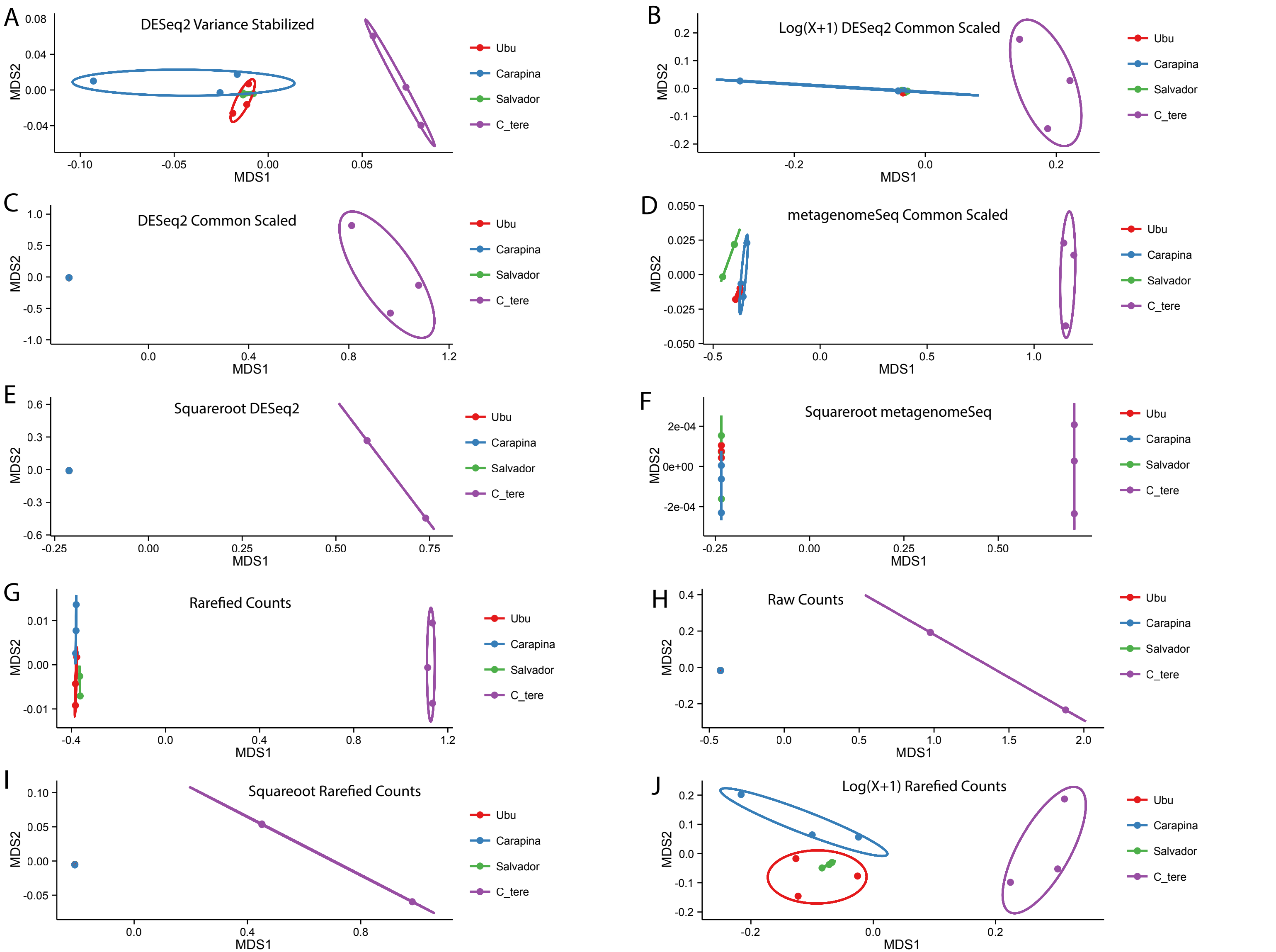

Supplement: S1 Fig — (A) OTU counts have been normalized using a variance stabilized approach as described in the main methods section. (B) A Log10(X+1) transformation on the DESeq2 common scaled OTU table, Love et al. (2014) stated that the variance stabilized normalization was similar to a log normalization [41]. (C) OTU counts have been normalized to a common scale using DESeq2. (D) OTU counts have been normalized using the cumulative-sum scaling method applied with the R package ‘metagenomeSeq’ [42]. (E) OTU counts have been normalized to a common-scale using DESeq2 and square root transformed. (F) OTU counts have been normalized using cumulative-sum scaling and square root transformed. (G) OTU counts have been randomly subsampled without replacement to 100,000 sequences per sample. (H) Raw, non-tranformed and non-normalized, OTU counts. (I) Subsampled OTU counts have been square root transformed. (J) Subsampled OTU counts have been log10(X+1) transformed. (PDF) [file pone.0132248.s001.pdf]

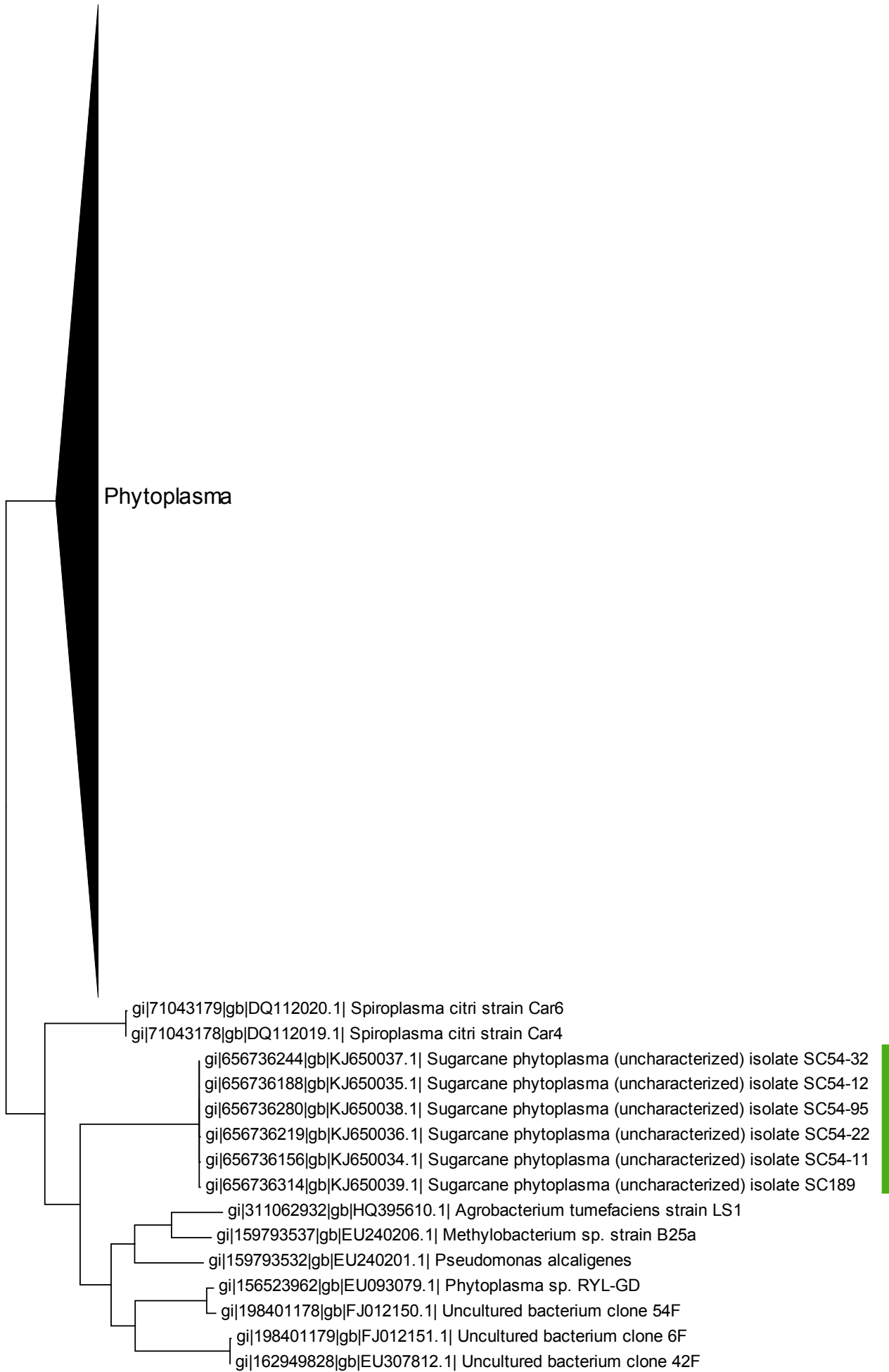

Plastid Sequences

0.05

Supplement: S2 Fig — As described in the methods section, the GenBank nucleotide database was screened for any sequence affiliated with the SSU rRNA gene from Phytoplasma spp. All sequences were aligned using PyNAST and a maximum likelihood tree was generated using MEGA v6. Misannotated sequences were excluded from the database. (PDF) [file pone.0132248.s002.pdf]
